# Supplementary material for: Nutritional deficiency in an intestine-on-a-chip recapitulates injury hallmarks associated with environmental enteric dysfunction
Source: Nat Biomed Eng. 2022 Jun 23;6(11):1236–47. doi: 10.1038/s41551-022-00899-x (PMC9652151; doi:10.1038/s41551-022-00899-x)
Supplement: Supplementary file 1 — Supplementary figures. [file 41551_2022_899_MOESM1_ESM.pdf]

nature portfolio

https://doi.org/10.1038/s41551-022-00899-x

Supplementary information

Nutritional deficiency in an intestine-on-a-chip recapitulates injury hallmarks associated with environmental enteric dysfunction

In the format provided by the authors and unedited

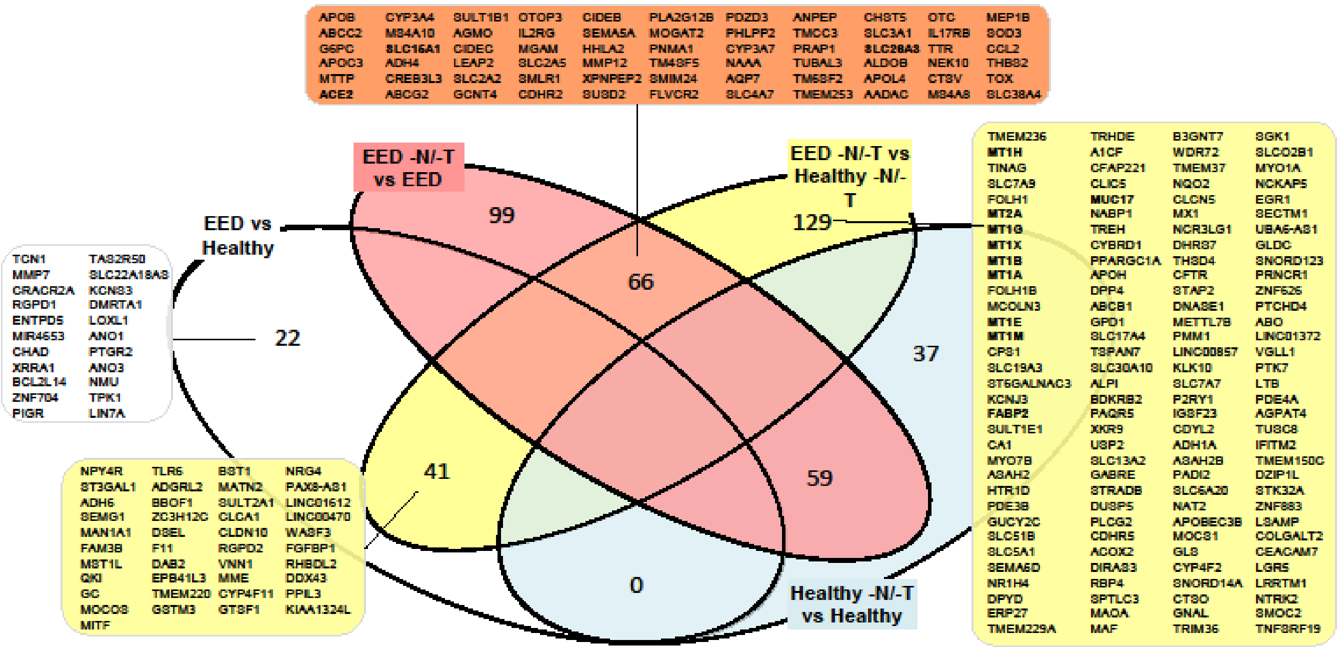

Supplementary Fig. 1 l A Venn diagram representation showing overlap of differentially regulated genes ($p < 0.01$; fold change > 2) for EED and Healthy Chips exposed to complete and nutritionally deficient media. Affected genes are listed and genes that were also differentially expressed in the clinical EED gene signature are in bold. n = 3 chips for each condition. Each chip = One biological replicate.

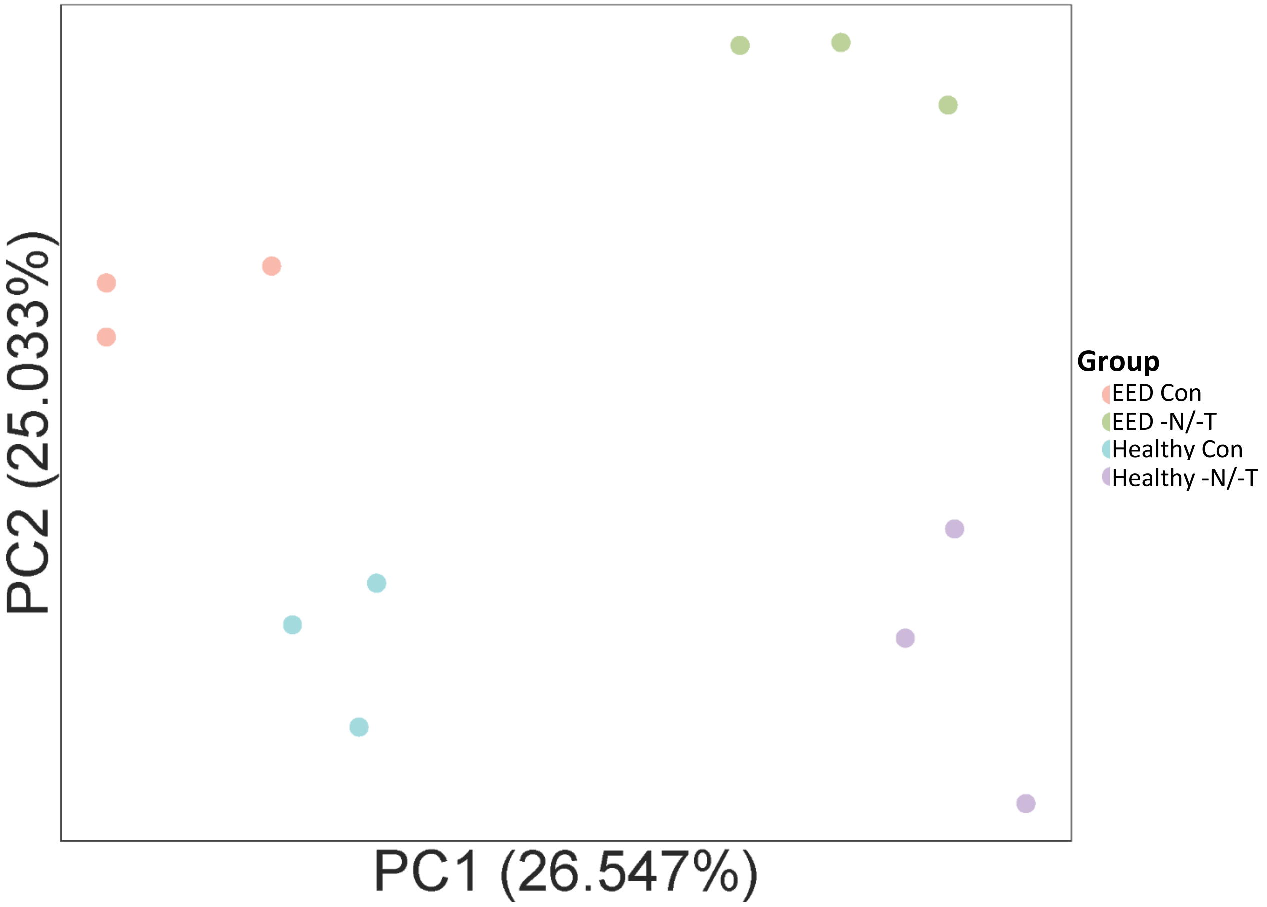

Supplementary Fig. 2 l Principal component analysis of transcriptomes from the different experimental groups showing uniformity of samples in each group as well as similarity and difference between the chip models. n = 3 chips for each condition. Each chip = One biological replicate.

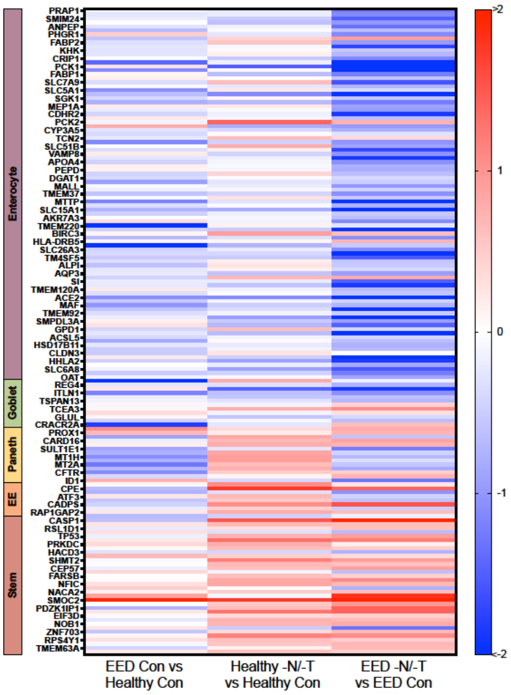

Supplementary Fig. 3 I Heatmap showing expression levels of previously identified small intestine cell-type markers (red, upregulation; blue, downregulation; Enterocyte = enterocytes; Goblet, goblet cells; Paneth, Paneth cells; EE, enteroendocrine cells; Stem, stem cell). n=3 chips per condition. Each chip = One biological replicate. EED Chips express lower levels of enterocyte markers when compared to Healthy Chips in control medium and further downregulate these markers upon exposure to nutritional deficiency. Paneth cell markers are also notably decreased in EED Chips compared to Healthy Chips in control medium. Upon exposure to nutritional deficiency, Paneth cell markers were upregulated in Healthy Chips but downregulated in EED Chips. Stem cell markers were generally upregulated by Heathy and EED Chips upon exposure to nutritional deficiency.

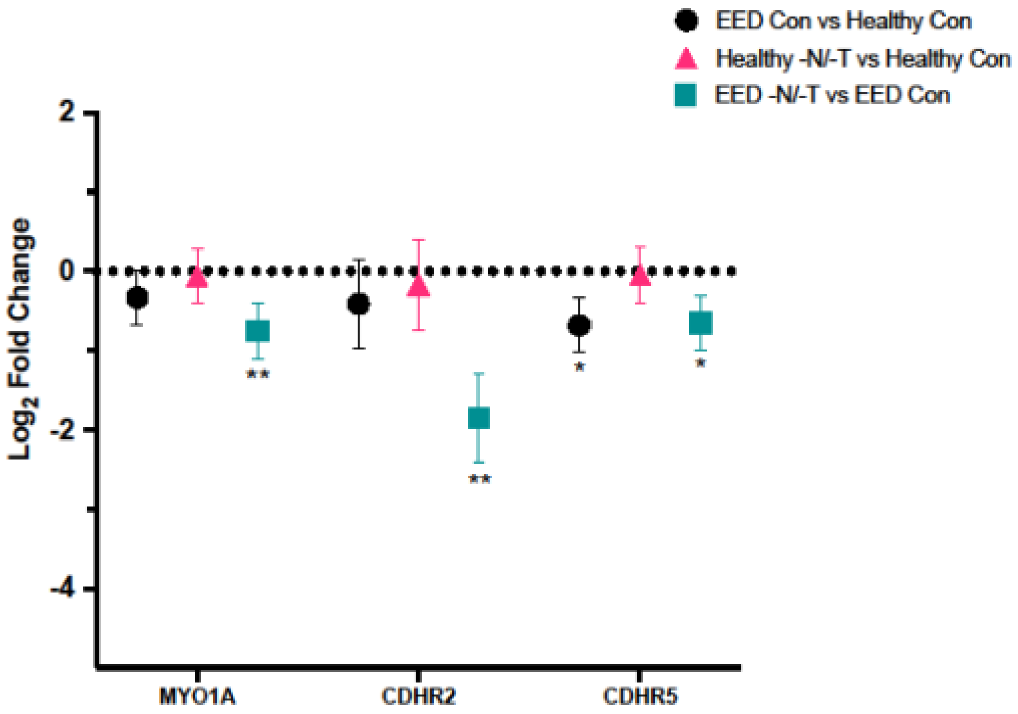

Supplementary Fig. 4 l Transcriptional pathway analysis revealed a strong theme of downregulation for genes related to brush border localization and structural integrity when EED Chips were exposed to -N/-T media. This included a 3.6-fold downregulation of CDHR2 ($q = 0.001$) and more modest downregulation of MYO1A (1.7-fold, $q = 0.0080$) and CDHR5 (1.6-fold, $q = 0.0187$), which are shown as the log2 fold change between the average expression levels along with the associated 95% confidence interval. n = 3 chips for each condition. Each chip = One biological replicate. *, $p < 0.05$; **, $p < 0.01$

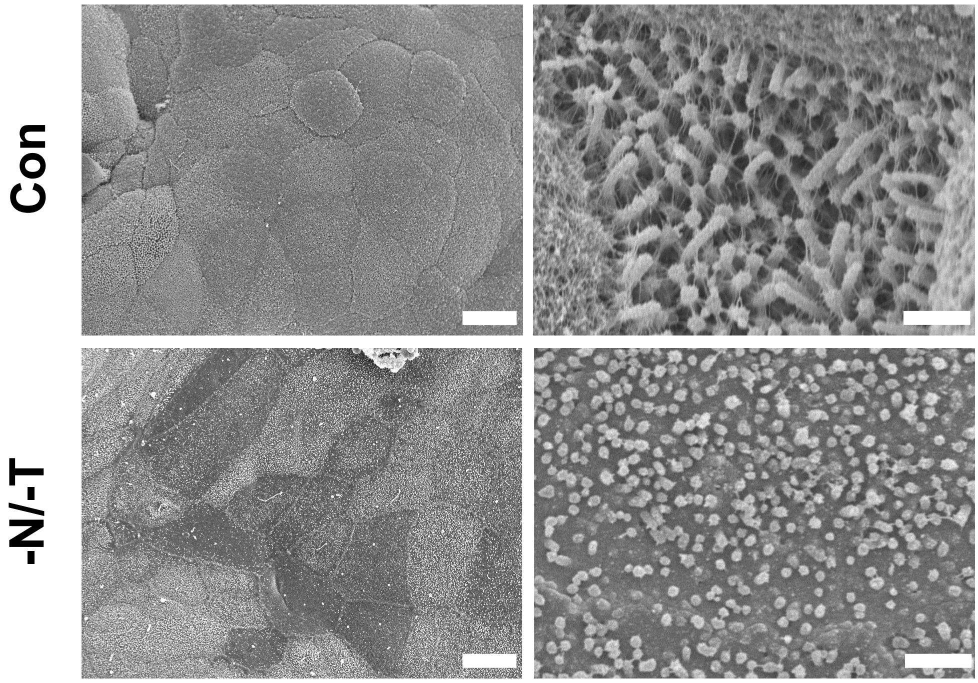

Supplementary Fig. 5 l SEM views of the apical surface in the Intestine Chip comparing the morphology of Control and -N/-T treated Healthy chips. Scale bar, 20 µm (left) and 2 µm (right).

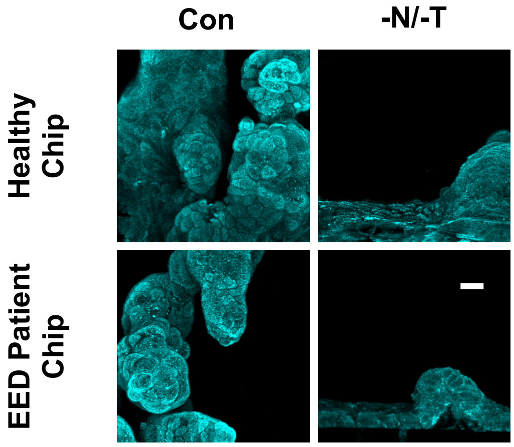

Supplementary Fig. 6 l Immunofluorescence microscopic view of a cross section through the intestinal epithelium on-chip stained for Apo B (Cyan); bar, 20 um.

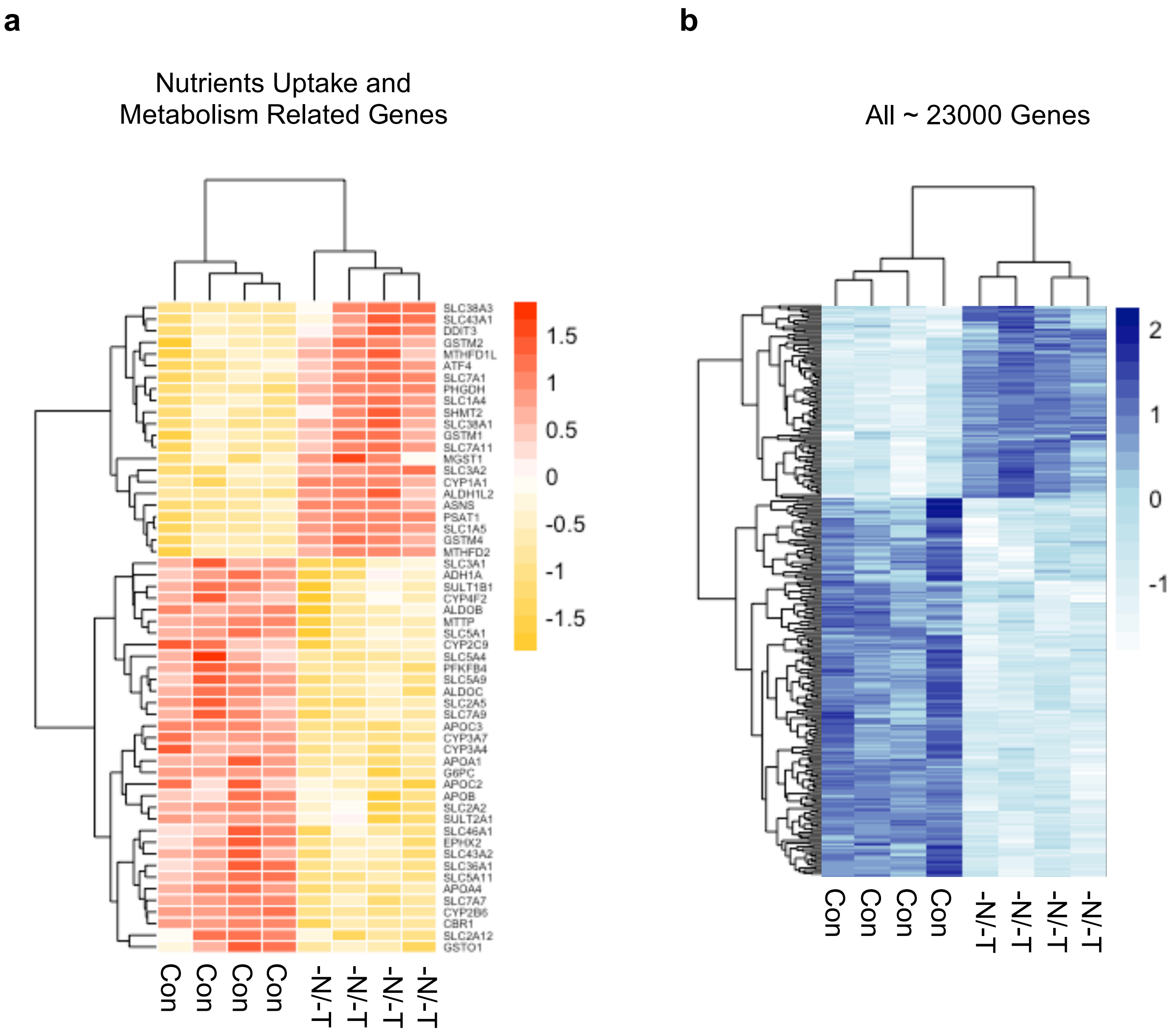

Supplementary Fig. 7 I a, Heatmap showing up and down regulated nutrients uptake and metabolism related genes in Control and -N/-T Healthy Chips. **b,** Heatmap showing all ~ 23000 up and down regulated genes in control and -N/-T healthy chips. n = 4 chips for each condition. Each chip = One biological replicate.

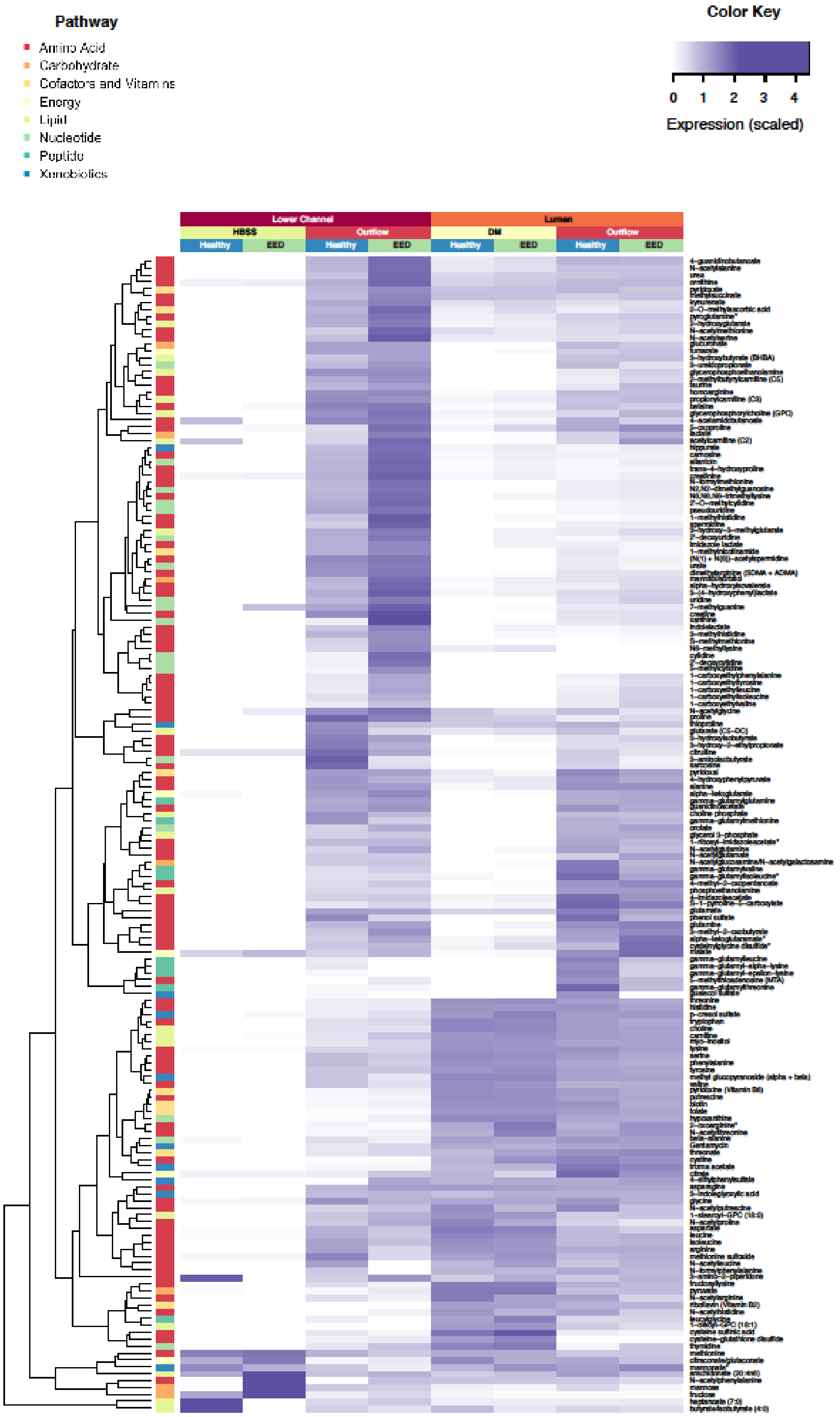

Supplementary Fig. 8 I Heatmap showing 167 metabolites found to be at higher abundance in the lower channel outflow vs lower channel inlet (DM, differentiation medium). n = 3 chips for each condition. Each chip = One biological replicate.

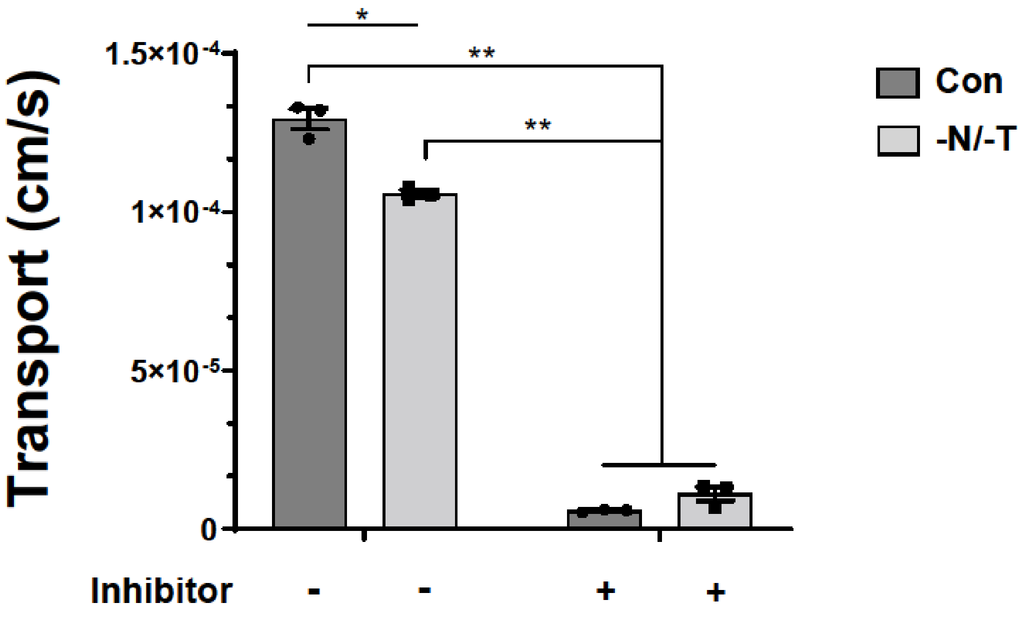

Supplementary Fig. 9 l Calculated transport of Gly-Sar dipeptide transfer from the lumen to the lower channel showing differences between Con and -N/-T, in Healthy Intestine Chips, in the presence or absence of Gly-Gly inhibitor. Con vs -N/-T, $p = 0.0022$, with vs without inhibitor $p = 0.000003$. n = 3 chips for each condition. Each chip = One biological replicate.
