# Supplementary material for: Nutritional deficiency in an intestine-on-a-chip recapitulates injury hallmarks associated with environmental enteric dysfunction
Source: Nat Biomed Eng. 2022 Jun 23;6(11):1236–47. doi: 10.1038/s41551-022-00899-x (PMC9652151; doi:10.1038/s41551-022-00899-x)
Supplement: Supplementary file 2 — Reporting Summary [file 41551_2022_899_MOESM2_ESM.pdf]

nature portfolio

Corresponding author(s): Donald E. Ingber

Last updated by author(s): May 8, 2022

Reporting Summary

Nature Portfolio wishes to improve the reproducibility of the work that we publish. This form provides structure for consistency and transparency in reporting. For further information on Nature Portfolio policies, see our Editorial Policies and the Editorial Policy Checklist.

Statistics

For all statistical analyses, confirm that the following items are present in the figure legend, table legend, main text, or Methods section.

n/a	Confirmed	
☐	☒	The exact sample size (*n*) for each experimental group/condition, given as a discrete number and unit of measurement
☐	☒	A statement on whether measurements were taken from distinct samples or whether the same sample was measured repeatedly
☐	☒	The statistical test(s) used AND whether they are one- or two-sided<br>*Only common tests should be described solely by name; describe more complex techniques in the Methods section.*
☐	☒	A description of all covariates tested
☐	☒	A description of any assumptions or corrections, such as tests of normality and adjustment for multiple comparisons
☐	☒	A full description of the statistical parameters including central tendency (e.g. means) or other basic estimates (e.g. regression coefficient) AND variation (e.g. standard deviation) or associated estimates of uncertainty (e.g. confidence intervals)
☐	☒	For null hypothesis testing, the test statistic (e.g. *F*, *t*, *r*) with confidence intervals, effect sizes, degrees of freedom and *P* value noted<br>*Give P values as exact values whenever suitable.*
☒	☐	For Bayesian analysis, information on the choice of priors and Markov chain Monte Carlo settings
☒	☐	For hierarchical and complex designs, identification of the appropriate level for tests and full reporting of outcomes
☒	☐	Estimates of effect sizes (e.g. Cohen's *d*, Pearson's *r*), indicating how they were calculated

Our web collection on statistics for biologists contains articles on many of the points above.

Software and code

Policy information about availability of computer code

Data collection	Microsoft Excel, PRISM-ImageJ (FIJI), Metabolon.
Data analysis	IMARIS (IMARIS 7.6 F1 workstation; Bitplane Scientific Software)<br>ImageJ (FIJI)<br>Graphpad Prism software<br>R/R Studio: A language and environment for statistical computing (packages: SCAN.UPC, limma, heatmaply)<br>COmprehensive Multi-omics Platform for Biological InterpretatiOn (COMPBIO)<br>Metabolon Visual PHIL

For manuscripts utilizing custom algorithms or software that are central to the research but not yet described in published literature, software must be made available to editors and reviewers. We strongly encourage code deposition in a community repository (e.g. GitHub). See the Nature Portfolio guidelines for submitting code & software for further information.

Data

Policy information about availability of data

All manuscripts must include a data availability statement. This statement should provide the following information, where applicable:

- Accession codes, unique identifiers, or web links for publicly available datasets
- A description of any restrictions on data availability
- For clinical datasets or third party data, please ensure that the statement adheres to our policy

The main data supporting the results in this study are available within the paper and its Supplementary Information. The organ-chip microarray data are available

from the Gene Expression Omnibus (GEO) database, under accession number GSE202282. The clinical mRNASeq data referenced as a comparison are available from GEO under accession number GSE159495. The metabolomics data are available from github at https://github.com/ranikay/eed-metabolomics-analysis.

Field-specific reporting

Please select the one below that is the best fit for your research. If you are not sure, read the appropriate sections before making your selection.

☒ Life sciences ☐ Behavioural & social sciences ☐ Ecological, evolutionary & environmental sciences

For a reference copy of the document with all sections, see nature.com/documents/nr-reporting-summary-flat.pdf

Life sciences study design

All studies must disclose on these points even when the disclosure is negative.

Sample size	Initial microarray experiments were carried out using one healthy donor (3 biological replicates) and one EED donor (3 biological replicates), and were reflective of a recent clinical EED transcriptomic signature derived from a larger population (SEEM study, with 25 healthy donors and 52 EED donors). Subsequent validation studies were carried out using 1–3 healthy donors (3–9 biological replicates) and 1–2 EED donors (3–8 biological replicates) per experiment (details provided in Methods and in figure legends). Sample sizes were determined on the basis of previous experimental experience as well as of the power of the statistical test performed.
Data exclusions	No data were excluded from the analyses.
Replication	Microarray sequencing data and metabolomics were done once with 3 biological replicates. All experiments were reproducible, as assessed in multiple intestine-chip culture devices per experiment. We did not have cases of irreproducibility.
Randomization	The intestine chips were randomly allocated into the experimental groups.
Blinding	The investigators were blinded to group allocation, as the chips were in random order when numbered at the time of seeding and placed in groups based on chip number. For other data collection and analysis, the investigators were not blinded to the identities of the samples. However, all experimental and control samples were collected and analysed at the same time under the same conditions, and the quantification was carried out with the same software settings.

Reporting for specific materials, systems and methods

We require information from authors about some types of materials, experimental systems and methods used in many studies. Here, indicate whether each material, system or method listed is relevant to your study. If you are not sure if a list item applies to your research, read the appropriate section before selecting a response.

Materials & experimental systems

n/a	Involved in the study	
☐	☒	Antibodies
☒	☐	Eukaryotic cell lines
☒	☐	Palaeontology and archaeology
☒	☐	Animals and other organisms
☐	☒	Human research participants
☒	☐	Clinical data
☒	☐	Dual use research of concern

Methods

n/a	Involved in the study	
☒	☐	ChIP-seq
☒	☐	Flow cytometry
☒	☐	MRI-based neuroimaging

Antibodies

Antibodies used	Primary antibody: Rabbit polyclonal to Apolipoprotein B (1:100, Abcam ab20737).<br><br>Secondary antibody: Donkey anti-Rabbit IgG (H+L) Cross-Adsorbed Secondary Antibody, DyLight 488 (1:1500; ThermoFisher SA5-10038)<br><br>Other labelling: Alexa Fluor 568 Phalloidin (Invitrogen A12380) and Wheat Germ Agglutinin, Alexa Fluor 488 Conjugate (ThermoFisher W11261)
Validation	Apolipoprotein B: recommended for immunofluorescence staining by the provider. We performed control experiments, including no primary antibody (negative) controls and comparison to experimental-group staining patterns. Additionally, this antibody has been cited in the literature many tens of times, as listed on the manufacturer's website.

Human research participants

Policy information about studies involving human research participants

Population characteristics	Healthy cells isolated from human intestinal organoids were derived from biopsy specimens from the grossly uninflamed duodenum of pediatric patients (2–11 years old) diagnosed with ulcerative colitis during an endoscopy procedure.<br><br>EED cells isolated from human intestinal organoids were derived from biopsy specimens collected during an endoscopy procedure in malnourished pediatric patients (1.5–1.9 years old, male) diagnosed with EED.
Recruitment	Healthy duodenal biopsies were derived from biopsied tissue of a patient undergoing endoscopy for gastrointestinal complaints.<br><br>EED duodenal biopsies were derived from patients in the previously published Study of Environmental Enteropathy and Malnutrition (SEEM). The patients were recruited at birth and followed to 24 months of age. Duodenal tissue biopsies were collected during and endoscopy procedure in patients who failed to respond to nutritional intervention.
Ethics oversight	For the healthy duodenal biopsies, informed consent and developmentally appropriate assent were obtained at Boston Children's Hospital from the donors' guardian and the donor, respectively. All methods were carried out in accordance with the Institutional Review Board of Boston Children's Hospital (Protocol# IRB-P00000529) approval.<br><br>For the EED duodenal biopsies, informed consent was obtained at the housedhold level in a rural district of Matiari, Sind, Pakistan from the donors' guardians. All methods were carried out in accordance with the AKU Ethical Review Committee's approval (ERC number 3836-Ped-ERC-15).

Note that full information on the approval of the study protocol must also be provided in the manuscript.
